# Analysis of the causes of recurrence after frontalis suspension using silicone rods for congenital ptosis

**DOI:** 10.1371/journal.pone.0171769

**Published:** 2017-02-16

**Authors:** Chang Yeom Kim, Byeong Jae Son, Jangyup Son, Jongill Hong, Sang Yeul Lee

**Affiliations:** 1 The Institute of Vision Research, Department of Ophthalmology, Yonsei University College of Medicine, Seoul, Korea; 2 Department of Ophthalmology, Kim’s Eye Hospital, Myung-Gok Eye Research Institute, Konyang University College of Medicine, Seoul, Korea; 3 Department of Ophthalmology, School of Medicine, Kyungpook National University, Daegu, Korea; 4 Department of Materials Science and Engineering, Yonsei University, Seoul, Korea; 5 Mechanical Science and Engineering, University of Illinois at Urbana-Champaign (UIUC), Urbana, Illinois, United States of America; 6 Lee’s Eye Clinic, Seoul, Korea; Universita degli Studi di Roma La Sapienza Facolta di Medicina e Psicologia, ITALY

## Abstract

**Background:**

Silicone rod is a commonly used synthetic suspension material in frontalis suspension surgery to correct blepharoptosis. The most challenging problem and a decisive drawback of the use of silicone rod is a considerable rate of ptosis recurrence after surgery. We examined patients with recurred ptosis and assessed the physical and micromorphological properties of implanted silicone rods to determine the causative mechanisms of recurred ptosis after frontalis suspension using silicone rod.

**Methods:**

This is a prospective observational case series of 22 pediatric patients with recurred ptosis after frontalis suspension using silicone rods for congenital ptosis. Implanted silicone rods were observed and removed during the operation for correction of recurred ptosis. The removed silicone rods were physically and micromorphologically evaluated to determine the cause of recurrence.

**Results:**

Pretarsal fixation positions migrated upward, whereas suprabrow fixation positions migrated downward during ptosis recurrence. The breaking strength of implanted silicone rods was reduced by approximately 50% during 3 years. Cracks, debris, and loss of homogenous structure with disintegration were observed on scanning electron micrographs of implanted silicone rods in patients with recurred ptosis. Preoperative severe degree of ptosis also contributed to recurred ptosis.

**Conclusions:**

Recurrence of ptosis after frontalis suspension using silicone rod was associated with physical changes of implanted silicone rods, including positional migration, weakened tensile strength, and micromorphological changes in combination with patients’ characteristics.

## Introduction

Blepharoptosis is one of the most common oculoplastic disorders, and different surgical techniques have been described to correct it. Maximal/super-maximum levator resection, Whitnall’s sling, or check ligament suspension was introduced for severe ptosis with poor levator muscle function (LF) [1–4], however, frontalis suspension is a commonly used surgical method for patients with congenital ptosis and poor LF [5–7]. Many suspension materials are available, including autogenous or banked fascia lata and synthetic materials such as silicone rod, monofilament nylon, polyfilament nylon, expanded polytetrafluoroethylene (ePTFE), and polypropylene [7–11]. Although autogenous fascia lata is considered the best suspension material due to its lower rates of recurrence and complications [6,7,10–12], and it remains an excellent choice for severe congenital ptosis [13], synthetic materials may be preferred in children younger than 3 years or for patients who do not want an additional harvesting operation. Meanwhile, some reports have indicated that autogenous fascia and alloplastic materials resulted in similar functional and cosmetic results in frontalis suspension surgery [8,9]. In addition, a recent review study suggested that PTFE is the material with lowest recurrence rates as well as good cosmetic and functional results [7].

Frontalis suspension using silicone rod is a safe, simple, easy, and effective surgical procedure [14,15]. Silicone rod has elastic properties, which preserves eyelid closure function and results in lower degree of lagophthalmos [16]. Therefore, it is commonly used in young children or patients who have a high risk of corneal exposure, such as those with myasthenia gravis, chronic progressive external ophthalmoplegia, or inadequate Bell’s phenomenon [10,15]. However, recurred ptosis is the most challenging problem in silicone rod suspension surgery, and limits its use to temporary treatment [8,9,17,18].

The purpose of this study was to examine patients with recurred ptosis after frontalis suspension using silicone rod, and to investigate the physical and micromorphological properties of implanted silicone rods to determine the causative mechanisms of recurred ptosis after frontalis suspension using silicone rod.

## Materials and methods

This is a prospective observational case series. Yonsei University Health System, Severance Hospital, Institutional Review Board (IRB) / Ethics Committee approval was obtained for this study. The study adhered to the tenets of the Declaration of Helsinki, and written informed consent was obtained from all participants (parents or legal guardians).

Twenty-two pediatric patients with recurred congenital ptosis were enrolled in this study. They had severe congenital ptosis with poor LF (LF ≤ 4 mm). They did not have neuromuscular diseases, blepharophimosis, Marcus Gunn jaw-winking, or strabismus.

Over the past two decades, frontalis suspension surgery was our preferred surgical method for patients with severe ptosis with poor LF. Therefore, patients who were younger than the age of 3 years were given their first frontalis suspension surgery using silicone rods (Frontalis Suspension Set #585192; Beaver-Visitec International, Inc., Waltham, MA, USA), as they were too young to have their autogenous fascia lata extracted. Since the first surgery, patients received regular postoperative follow-up care. After reaching the age of 3 years, patients underwent a second operation for recurred ptosis using direct tarsal and frontalis fixation with autogenous fascia lata, as described by Spoor [19]. Due to young patients’ tendency to be uncooperative, all surgeries were performed under general anesthesia by the same surgeon (SYL) from March 2006 to December 2013.

Preoperative degree of ptosis, LF, and amount of eyelid lift during the first operation were reviewed from the medical records. Recurrence was defined as re-drooping of the eyelid that was clearly observed by both the guardians and physicians of patients. During the second operation, the previously implanted silicone rod was observed in situ and then removed, and the frontalis suspension procedure was repeated.

### Surgical procedures

Frontalis suspension using silicone rod was performed in the pentagonal technique of Fox [5]. The silicone rod was passed through two eyelid stab incisions and fixed at the pretarsal tissue with 6–0 polypropylene sutures. Then, the silicone rod was passed under the orbital septum to the brow in a pentagonal pattern. After adjusting the palpebral fissure width (PF), the silicone rod was fixed using a silicone sleeve and an anchoring 6–0 polypropylene suture at the central suprabrow stab incision site.

### Measurement of change in fixation position

The positional changes of sleeve and pretarsal fixation of the implanted silicone rod were observed. The position of the previously implanted silicone sleeve was identified by palpation, and the distance between the position of the silicone sleeve and the central suprabrow scar was measured. Then, a skin incision was performed along the eyelid crease and careful dissection was carried out to find the previously implanted silicone rod. The distance between the previous eyelid stab incision level and the lowest position of the migrated silicone rod was measured. A skin incision was also created at the suprabrow scar, and the status of the silicone sleeve and anchoring suture were observed. Then, the silicone sleeve and rod were carefully removed from the central suprabrow site so as to minimize damage to them. The removed silicone rod was divided into two pieces; one piece was used for a destructive pull test to measure breaking strength, and the other was used for micromorphological examination.

### Measurement of tensile strength and breaking strength

**Fig 1:**
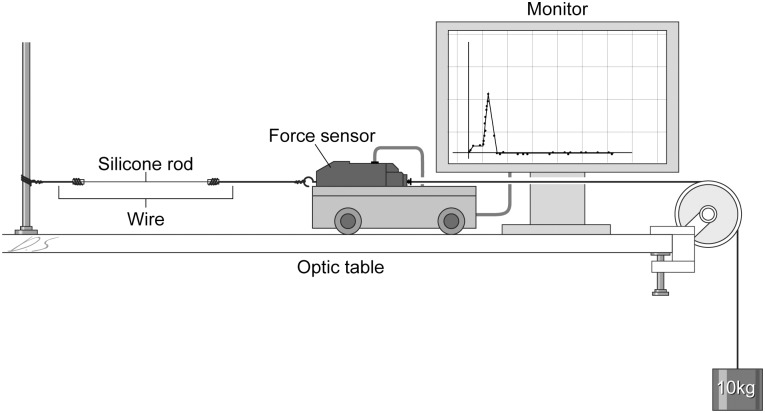
Measurement of breaking strength of implanted silicone rods using the force sensor system.

A destructive pull test was performed to measure the tensile strength of the implanted silicone rod and an unused silicone rod (Fig 1). The force sensor (the PASCO CI-6746 Economy Force Sensor, PASCO scientific^®^, Roseville, CA, USA) on a cart was placed on the optic table. The silicone rod was cut to 2 cm length, and both ends were connected to the strings. One string was attached to the fixed stand, and the other was attached to a hanging mass of 10 kg weight passed through a pulley. The cart/force sensor was pulled by the string attached to the hanging mass, and then the cart accelerated toward the pulley. The silicone rod segment was pulled from both sides and was severed by the force. The tensile strength was recorded by a computer interface connected to the force sensor. The maximal destructive force was measured when the silicone rod broke, and the tension at that point was the breaking strength.

### Micromorphological analysis

The harvested segment of the silicone rod was air-dried and sputter-coated (ion sputter E1010, Hitachi, Tokyo, Japan) for 6 minutes. Then, it was examined and photographed with a scanning electron microscope (FE SEM S-800, Hitachi, Tokyo, Japan) at the acceleration voltage of 20 kV. The surface and cross-section of the silicone rod were examined by a specialist that was blinded to the sample identity. An unused silicone rod segment was examined as the control. The observed micromorphological changes were categorized as mild (grade 0), moderate (grade 1), or severe (grade 2) based on previously published standards (Table 1) [20].

**Table 1:** Micromorphological grades of implanted silicone rod, harvested from patients with recurred ptosis.

Grade	Surface	Cross-section
Mild (0)	Only rough texture and no noticeable surface crack	Homogenous structure and retained original round form
Moderate (1)	Some cracks and debris	Homogenous structure with some internal changes
Severe (2)	Multiple cracks and much debris	Irregular and disintegrated structure

### Statistical analysis

The relationships between the physical and micromorphological changes in implanted silicone rods, the clinical features of patients including the degree of ptosis, the amount of intraoperative eyelid lift, duration of implanted silicone rod before corrective surgery, and the time of recurrence or the degree of eyelid re-drooping were analyzed. Two-sided statistical analyses were performed with the significance level set at 0.05, and using IBM^®^ SPSS^®^ Statistics version 20 software.

## Results

A total of 28 eyes of 22 patients were assessed in this study. Noticeable eyelid re-drooping developed approximately 30 months after silicone rod suspension surgery, and silicone rods had been implanted for approximately 37 months. Patients’ characteristics are presented in Table 2.

**Table 2:** Patients’ characteristics.

Patients (eyes)	22 (28)
Age (years)	
at silicone rod suspension	1.23 ± 0.81 [0.6, 2.9]
at silicone rod removal	4.33 ± 1.11 [3.2, 7.7]
Sex (number of eyes, male: female)	23: 5
Laterality (number of patients, unilateral: bilateral)	16: 6
Preoperative palpebral fissure width (mm)	
at silicone rod suspension	3.45 ± 1.51 [0.0, 6.0]
at silicone rod removal	5.36 ± 1.54 [2.5, 7.5]
Amount of eyelid lift during silicone rod suspension (mm)	4.09 ± 0.93 [2.5, 6.5]
Degree of re-drooping^a^ (mm)	2.13 ± 1.42 [0.0, 6.0]
Recurrence time^b^ (months)	29.32 ± 12.47 [3.0, 55.0]
Implantation duration of silicone rod (months)	37.24 ± 11.76 [7.0, 64.7]

Presented with Mean ± SD [min, Max].

^a^Measured shortly before silicone rod removal and autogenous fascia lata suspension.

^b^Time interval between silicone rod suspension and recurrence.

### Physical changes of implanted silicone rods

**Fig 2:**
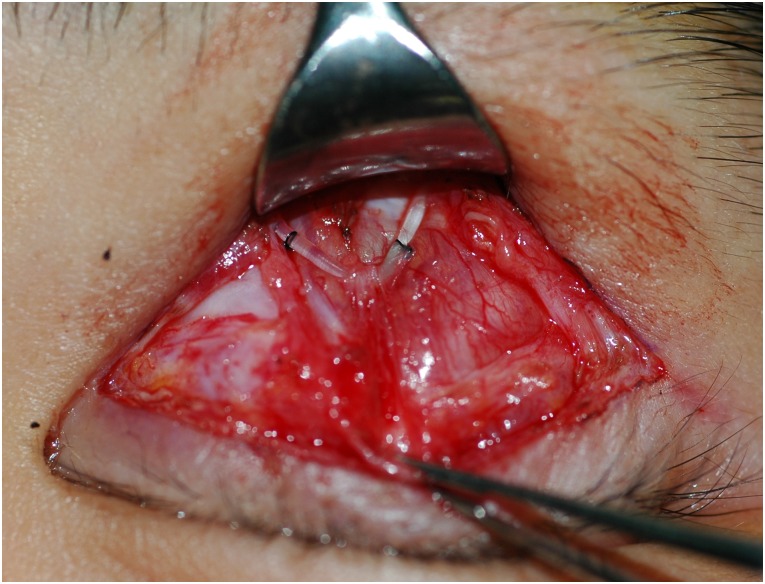
Intraoperative finding of implanted silicone rod. The pretarsal fixation sutures of implanted silicone rod migrated upward.

Pretarsal fixation sutures were observed for 21 eyes (75%), but all sutures were displaced upward (Fig 2). Above the eyebrow, silicone rods were positioned securely in the sleeves, and fixation sutures remained, but were displaced downward in all eyes. The pretarsal and brow fixation positions migrated 6.17 ± 2.50 mm upward and 4.24 ± 1.23 mm downward, respectively (Table 3).

**Table 3:** Physical changes of implanted silicone rods.

**Migration of position (mm)**		
Upward from pretarsal fixation	6.17 ± 2.50	
Downward from suprabrow fixation	4.24 ± 1.23	
**Breaking strength (N)**		**P-value**
Unused silicone rod	5.41	
Used silicone rod	2.88 ± 0.38	
Implantation duration (months)		
< 36	2.91 ± 0.35	0.76
≥ 36	2.85 ± 0.43	
Amount of intraoperative eyelid lift (mm)		
< 5.0	2.92 ± 0.38	0.27
≥ 5.0	2.60 ± 0.32	
Degree of re-drooping (mm)		
< 3.0	2.98 ± 0.36	0.053
≥ 3.0	2.57 ± 0.25	

Presented with Mean ± SD (standard deviation) [min, Max].

**Fig 3:**
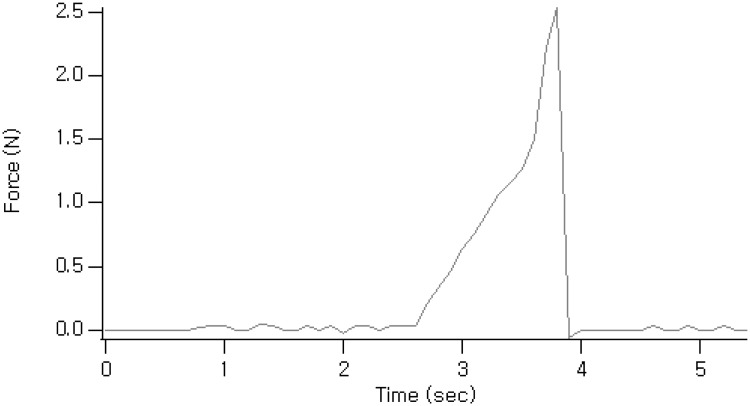
Representative creep curve of implanted silicone rod.

A representative creep curve of the silicone rod is shown in Fig 3. Breaking strength of the implanted silicone rod ranged from 2.37 to 3.51 N, with a mean strength of 2.88 N, which was approximately 53.2% of the unused silicone rod strength of 5.41 N (Cross-sectional area = 0.2025π㎟, Table 3). Silicone rods removed from eyes with ≥ 3.0 mm of re-drooping had breaking strengths that were lower than those removed from eyes with < 3.0 mm of re-drooping (*P* = 0.053). There was a significant correlation between the degree of eyelid re-drooping and breaking strength (*P* = 0.02, Pearson correlation coefficient = −0.58).

### Micromorphological changes of implanted silicone rods

**Fig 4:**
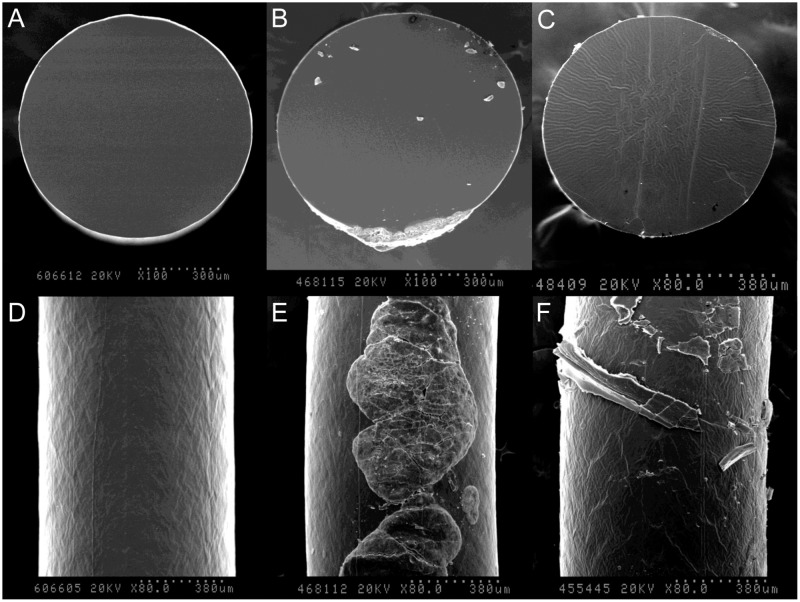
Micromorphological analysis of unused and implanted silicone rods. (A, B, C) Surface structure, (D, E, F) cross-sectional structure, (A, D) unused, (B, C, E, F) implanted silicone rods.

Unused silicone rods had smooth surfaces and the cross-sections revealed homogenous, clean structures internally. However, used silicone rods displayed cracks and debris on the surface, and the cross-sections revealed loss of homogenous structures and marked disintegration (Fig 4).

**Fig 5:**
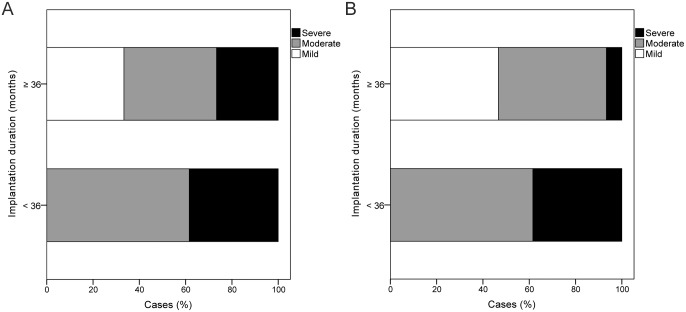
Micromorphological changes of silicone rods with respect to the implantation duration. (A) Surface structure, (B) cross-sectional structure.

Moderate to severe changes in surface properties and in cross-sectional micromorphologies were observed in 82.1% and 75%, respectively, of implanted silicone rods. The grades of surface changes were significantly correlated with the grades of cross-sectional changes (*P* < 0.01). There were no significant differences in micromorphological changes of implanted silicone rods between one patient had earlier recurrence than the average recurrence time (30 months) and the others (*P* = 0.80 for surface changes, *P* = 0.74 for cross-sectional changes). However, more severe changes were observed in silicone rods implanted for less than 3 years (*P* = 0.07 for surface changes, *P* < 0.01 for cross-sectional changes) (Fig 5).

### Factors associated with recurred ptosis

Comparisons of the characteristics with respect to recurrence time < 30 months, overall average recurrence time (early group), and recurrence time ≥ 30 months (late group) are shown in Table 4. Fixation positions migrated further and breaking strength was slightly greater in the late recurrence group, but these differences were not statistically significant.

**Table 4:** Comparisons of the characteristics between early and late recurrence group^a^.

Recurrence time	< 30 months	≥ 30 months	P-value
Age at silicone rod suspension (months)	14.49 ± 11.41	14.75 ± 7.72	0.95
Amount of intraoperative eyelid lift (mm)	4.13 ± 1.14	4.04 ± 0.66	0.79
Migration of eyelid fixation position (mm)	5.61 ± 2.76	6.58 ± 2.31	0.39
Migration of brow fixation position (mm)	3.85 ± 0.97	4.59 ± 1.38	0.17
Breaking strength of implanted silicone rod (N)	2.80 ± 0.40	2.94 ± 0.37	0.49
Surface change (grade 0:1:2) ^b^	3:8:4	2:6:5	0.80
Cross-sectional change (grade 0:1:2) ^b^	3:9:3	4:6:3	0.74

^a^Recurrence time < 30 months, overall average recurrence time (early group), and recurrence time ≥ 30 months (late group).

^b^Micromorphological changes of implanted silicone rod on scanning electron microscopy.

Multiple linear regression analysis showed that female sex (unstandardized coefficients *B* = 58.44, *P* = 0.04), preoperative PF (*B* = 8.95, *P* = 0.046), and severe micromorphological change defined as the summation of surface and cross-sectional grades ≥ 3 (*B* = −38.45, *P* = 0.03) were statistically significant factors associated with recurrence time. The degree of eyelid re-drooping was significantly associated with severe micromorphological change (*B* = 1.92, *P* = 0.046) (Table 5).

**Table 5:** Results of multiple linear regression analysis for factors influencing recurrence time and the degree of re-drooping.

Variables	Recurrence time		Degree of re-drooping	
	B^a^	P-value	B^a^	P-value
Age at silicone rod suspension (months)	-0.87	0.16	0.06	0.29
Sex (male = 0, female = 1)	58.44	0.04	-3.34	0.11
Laterality (unilateral = 0, bilateral = 1)	-12.98	0.10	0.69	0.31
Preoperative PF (mm)	8.95	0.046	N/A	
Amount of intraoperative eyelid lift (mm)	N/A		0.76	0.10
Migration of eyelid fixation position (mm)	5.69	0.06	-0.29	0.25
Migration of brow fixation position (mm)	0.79	0.65	-0.37	0.09
Breaking strength of implanted silicone rod (% of control)	-168.59	0.06	0.40	0.94
Severe micromorphological change^b^	-38.45	0.03	1.92	0.046
Adjusted R^2^ of regression model	0.919		0.943	

Variables were selected for the best explanatory power without multicollinearity.

^a^Unstandardized coefficient B.

^b^Summation of surface and cross-sectional grades of implanted silicone rod ≥ 3 on scanning electron microscopy (adjusted as 1).

PF, palpebral fissure width; N/A, non-applicable.

## Discussion

A recent study evaluating the mechanical properties of synthetic suspension materials suggested that silicone rod had the most suitable mechanical properties for ptosis surgery because it would require a relatively low force to stretch with fairly reasonable work of fracture [21]. However, frontalis suspension using silicone rods is associated with some recurrence of ptosis, which causes surgeons to hesitate before using silicone rods. Reported recurrence rates ranged from 7–44% in frontalis suspension surgery using silicone rod [9,14,22,23]. Nucci et al. reported that the margin reflex distance was progressively reduced by 0.6 mm within the first 3 months after surgery, and a further reduction of 0.2 mm occurred 3–12 months after frontalis suspension surgery with silicone band [24]. Another study that was conducted on Korean pediatric patients who underwent essentially the same surgical procedure as that in the present study reported a recurrence rate of 29.2% in bilateral cases and 11.1% in unilateral cases 3 years after surgery [25].

There have been few studies on the causes of recurred ptosis after frontalis suspension surgery [10]. In this study, we evaluated patients with recurred ptosis and performed physical and micromorphological analyses of the implanted silicone rods to identify factors associated with ptosis recurrence after frontalis suspension surgery using silicone rod. To the best of our knowledge, this study is the first multifactorial investigation of silicone rod performance in frontalis suspension surgery.

Inadequate bond formation between the synthetic material and the surrounding tissue was suggested as a possible cause of recurred ptosis after frontalis suspension surgery [26]. Another possible cause of ptosis recurrence was suggested to be a cheese-wiring effect of the suspension material [10]. That study reported that silicone rods slid upward from the fixation sites at the tarsal plate, and that the recurrence rate was significantly lower when the silicone rod was sutured to the tarsal plate than when it was not sutured [10]. These results suggest that fixation of the rod to the tarsal plate may reduce the cheese-wiring effect and prevent the silicone rod from sliding before scar formation, which would reduce early postoperative recurrence [10].

We found that the previously implanted silicone rods migrated from their initial positions. Silicone rods fixed at pretarsal positions slid upward from the stab incision levels, whereas those fixed at eyebrow positions slid downward. Although there was no statistical significance, the migration distances from fixation positions were larger in the late recurrence group than in the early recurrence group, contrary to our expectations. This could be because implantation duration of silicone rod was longer in the late recurrence group, and the cheese-wiring effect that made implanted silicone rods slide was more pronounced as time passed.

Silicone rods have linear elastic behavior and reduce inevitable postoperative lagophthalmos after frontalis suspension surgery [10,15]. The implanted silicone rod is pulled by lasting stress due to gravity and tension of the orbicularis oculi muscle. The implanted silicone rod withstands loads that tend to elongate it, and eventually may be transformed. In our destructive pull test, the creep curve of strain versus time under constant stress and temperature displayed these effects. We observed that breaking strength was reduced from 5.41 N in unused silicone rods to 2.88 N in silicone rods approximately 3 years after implantation, and correlated with the degree of recurred ptosis. This indicates that implanted silicone rods undergo structural deformation in patients with recurred ptosis. Breaking strength was much lower in patients with more severe re-drooping. Breaking strength also tended to decrease when the amount of intraoperative eyelid lift was large, although this trend was not statistically significant. The results showed that higher stress loads caused greater structural deformation.

Silicone is a polydimethylsiloxane derivative and a nonabsorbable synthetic material, but in vivo degradation has been reported [27,28]. In this study, implanted silicone rod displayed disintegrative changes including multiple cracks, surface debris, and loss of regular homogenous structure. A previous study evaluating patients with recurred blepharoptosis after frontalis suspension with polyfilament nylon (Supramid Extra^®^, S. Jackson, Inc., Alexandria, VA, USA) reported morphological degradation of the nylon material [20]. The authors suggested this change of suspension material could be one of the possible cause of recurred ptosis. The micromorphological changes we observed in the implanted silicone rods were similar to those observed previously in the implanted Supramid Extra^®^; however, relatively minor changes were observed in silicone rod cross-sections, which may be attributed to the monofilament structure of silicone rod versus the polyfilament structure of the Supramid Extra^®^. In contrast with expectations and previous results for the Supramid Extra^®^ that longer implantation times were associated with greater micromorphological changes, more severe changes were observed in silicone rods implanted for less than 3 years. This result suggests that patients who had severe changes in implanted silicone rods could undergo early reoperation. Micromorphological changes in implanted silicone rods were not significantly different between cases with earlier recurrence than the mean recurrence time and those that had later recurrence. However, multiple linear regression analysis revealed that micromorphological changes did significantly affect both the recurrence time and the degree of eyelid ptosis after recurrence when the variable was adjusted for severe micromorphological change.

Recurrence occurred earlier in cases with more severe ptosis. It may have been contributed by the fact that patients with more severe ptosis needed more force against tension of orbicularis oculi muscle and gravity to lift drooped eyelid. Although the data appeared to suggest that recurrence time was longer in female patients, this difference with respect to sex could not have a significant meaning due to the shortage of female patients in this study.

There are limitations to this study that must be considered when evaluating the conclusions. The exact time of recurrence could not be determined because we could not meet the patients on a daily basis. There was discrepancy between recurrence time and implantation time because the corrective operation was performed electively after eyelid re-drooping occurred. Since the majority of patients enrolled in the study were too young to perform accurate measurement of LF, we could not find the role of LF in recurred ptosis. However, there was no great difference in LF because all patients had poor LF. The tensile strength of the implanted silicone rod could be influenced by the test environment. Implanted silicone rods may have been damaged during the surgical dissection procedure, which could change their mechanical and micromorphological properties. Different surgical methods, or patients from other racial backgrounds, may have different outcomes than those in the present study. We could not investigate implanted silicone rods in cases without recurred ptosis. However, a significant strength of this study is the multilateral investigation of recurred ptosis after frontalis suspension surgery.

## Conclusions

This study revealed that physical changes of implanted silicone rods including positional migration, weakened tensile strength, and changes in micromorphological properties, could cause recurrence after frontalis suspension using silicone rod for congenital ptosis. Strong fixation of the silicone rod, improvement of the silicone rod’s properties, and overcorrection in patients with more severe ptosis could help lower the recurrence rate after silicone rod suspension surgery in congenital ptosis.

## Supporting information

S1 Dataset Minimal dataset of this study. (XLSX)
